# Enhancement of loop mediated isothermal amplification's sensitivity and speed by multiple inner primers for more efficient identification of *Vibrio parahaemolyticus*

**DOI:** 10.1016/j.mex.2023.102328

**Published:** 2023-08-21

**Authors:** Aekarin Lamalee, Chartchai Changsen, Wansadaj Jaroenram, Sureemas Buates

**Affiliations:** a Department of Microbiology, Faculty of Science, Mahidol University, Bangkok 10400, Thailand.; b Bioengineering and Sensing Technology Research Team, National Center for Genetic Engineering and Biotechnology (BIOTEC), National Science and Technology Development Agency (NSTDA), Pathum Thani 12120, Thailand

**Keywords:** Multiple hybrid, inner primers (MHP)-LAMP, LAMP, *V. parahaemolyticus*, Rapid detection, Multiple hybrid, inner primers (MHP)-LAMP for rapid detection of *V. parahaemolyticus*

## Abstract

The modified loop-mediated isothermal amplification (LAMP), called multiple hybrid, inner primers (MHP)-LAMP, was developed to enhance the efficiency of the existing LAMP-based assay for *Vibrio parahaemolyticus* detection. The method was built on a conventional LAMP assay by employing 2 newly designed extra sets of primers to increase the initial binding sites of core primers on the *V. parahaemolyticus*’s *rpoD* gene from 8 to 12. With this strategy, the assay detection sensitivity was increased by 10 folds, with the detection limit (DL) approaching 100 copies of purified target genomic DNA (gDNA) as analyzed by real-time turbidity measurement and gel electrophoresis. The MHP also accelerated the rate of DNA amplification by 30%, rendering the assay faster. The MHP-LAMP assay did not cross- react with other pathogens, indicating that it was highly specific for *V. parahaemolyticus* detection. Whilst *V. parahaemolyticus* was used as a study model herein, our idea of using MHP to maximize assay sensitivity and speed is considered as a universal strategy that can be applied to enhance efficiency of LAMP-based assays for detecting any DNA and RNA of interest.

• The strategy of using multiple hybrid, inner primers (MHP) to enhance LAMP assay's efficiency was demonstrated with success.
• The MHP enhanced the sensitivity and speed of the existing LAMP assay, designed to detect *V. parahaemolyticus*, by 10 times and 30%, respectively.
• The proposed strategy can be applied to boost up any other LAMP-based assay's diagnostic performance.

## Graphical abstract

**Figure:**
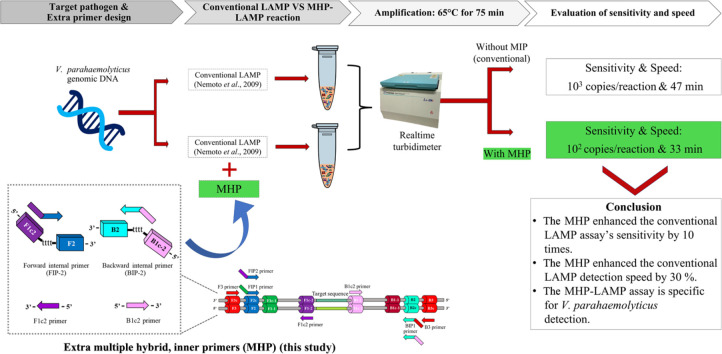

Specifications table

**Table:** 

Subject area:	Biochemistry, Genetics and Molecular Biology
More specific subject area:	Molecular Biology
Name of your method:	Multiple hybrid, inner primers (MHP)-LAMP for rapid detection of *V. parahaemolyticus*
Name and reference of original method:	• The conventional *rpoD* LAMP primers DOI: 10.4315/0362-028x.Jfp-10-519
Resource availability:	■ The MHP-LAMP experiments were performed with Loopamp Realtime Turbidimeter LA-320C. ■ All sets of extra primers were designed manually following the criteria suggested by A Guide to LAMP Primer Designing Manuals. (https://primerexplorer.jp/e/v5_manual/index.html). All designed primers were validated for cross dimerization by using Net Primer Design (Premier Biosoft).

## Introduction

*Vibrio parahaemolyticus* is a Gram-negative halophilic bacterium possessing a curved-rod shape with a single polar flagellum, belonging to the family of *Vibrionaceae*. This bacterium is commonly found in marine environment and coastal regions in topical and sub-tropical countries [1]. It is often related to living sea creatures including mussels, oysters, fish, prawns, and seaweed worldwide [2]. It is regarded as one of the major seafood-borne pathogens that cause outbreaks and sporadic cases of seafood-borne illness in humans globally [3]. Worldwide human gastroenteritis caused by *V. parahaemolyticus* infection is mostly associated with the consumption of raw or partially cooked contaminated seafood particularly shellfish, although wound exposure to contaminated water can also cause infection [1]. Based on the epidemiological report, the majority of outbreaks were happened following ingesting oysters and clams [4]. Moreover, this bacterium also caused economic burden in oyster aquaculture [5] and shrimp industry [6]. The proper seafood safety testing is the effective way to prevent seafood-borne illness caused by *V. parahaemolyticus*. Therefore, developing simple and sensitive diagnostic methods is essential.

For *V. parahaemolyticus* detection, a number of techniques are available from conventional culture-based methods to molecular-based methods. Generally, conventional culture-based methods are laborious and need 2-3 days to identify presumptive bacterial colonies with biochemical tests [7]. To date, molecular-based techniques including endpoint polymerase chain reaction (PCR) and quantitative real-time-PCR (qPCR) have been achieved for *V. parahaemolyticus* identification with high sensitivity and high specificity. However, these techniques require expensive equipment, reagents and well-trained staffs [7], [8], [9]. Loop-mediated isothermal amplification (LAMP) reported by Notomi et al. [10] is a promising candidate for *V. parahaemolyticus* detection [10,11]. This technique amplifies DNA under isothermal conditions by using a heat block or a water bath [10,12]. It is recognized for its high sensitivity, specificity, simplicity and rapidity. Therefore, it would be a magnificent tool to diagnose *V. parahaemolyticus*, a significant seafood-borne pathogen.

In this study, we developed a LAMP technique called multiple hybrid, inner primers (MHP)-LAMP, in which the target DNA was identified with high rapidity, sensitivity and efficiency. The MHP-LAMP method was successfully utilized for rapid and sensitive identification of *V. parahaemolyticus* by targeting *rpoD* (a housekeeping gene encoding for the principal RNA polymerase sigma factor) which is the species-specific gene of *V. parahaemolyticus* [13]. The MHP-LAMP sensitivity was validated by comparing it with that of a conventional LAMP assay and the MHP-LAMP specificity was validated with various non-related pathogens.

## Method details

### Bacterial strains, culture conditions and genomic DNA (gDNA) preparation

The reference strain of *V. parahaemolytius*, DMST 15285 (*tdh*^+^/*trh*^-^) acquired from the Department of Medical Science, Ministry of Public Health, Nonthaburi Province, Thailand was used as a positive control for primer optimization and sensitivity testing of the assay. The reference strain was grown in 5 mL tryptic soy broth (TSB) (Merch KGaA, Germany) supplemented with 2% NaCl (BDH, Poole, US) at 37 °C for 14 h in a shaking incubator at 250 rpm. A volume of 3 mL of the culture broth was transferred into a 1.5 mL microcentrifuge tube and centrifuged at 10,000 rpm for 2 min to remove the supernatant. The pellet was dissolved in Tris-EDTA (TE) buffer (Invitrogen, Grand Island, NY, USA) and used for gDNA extraction by GenUP^TM^ kit (Biotechrabbit, Berlin, Germany) following to the manufacturer's instruction. The gDNA concentration was quantified by a NanoDrop (DeNovix, DS-11 FX+, spectrophotometer/Fluorometer). The gDNA solution was aliquoted and stored at -80 °C.

### Primer design for multiple hybrid, inner primers (MHP)-LAMP

Six core primers targeting *rpoD* gene as described previously were used for generic *V. parahaemolyticus* detection herein [14]. They consisted of forward and backward inner primers 1: FIP1 and BIP1, forward and backward outer primers: F3 and B3, and forward and backward loop primers: LF and LB (Table 1). In addition to these primers, 4 extra ones (FIP2, BIP2, F1c-2 and B1c-2) locating between the FIP1 and BIP1 primers were designed in this study to further improve the reaction kinetics (Table 1, Fig. S1). The design was done manually following “A Guide to LAMP Primer Designing Manuals” with additional criteria to PCR primers as the following: (1) Guanine or cytidine at the 3′ end is preferred as they provide a more stable “clamped” target for the polymerase. (2) Gibbs free energy should be lower than −4 kcal/mol for both 5′- and 3′-ends. (3) Self-dimerization and cross-dimerization of more than four bases on each end are avoided as they highly promote self-polymerization leading to false positive amplification. These criteria were validated by using Net Primer Design (http://www.premierbiosoft.com).

**Table 1:** The *rpoD* primers and conditions used for MHP-LAMP amplification.

Primer	Sequence (5′ to 3′)	Length	Concentration (pmol/µL)	Refs.
Core primer				
FIP1 (F1c-1 + F2)	TGAATACGTCTAGCATCATTTCGTCGATCAATGAGTACGGCTACGA	46	20	[14]
BIP1 (B1c-1 + B2)	ACAGCAATGGATCGCGTTCCGATTTCTTCGGCATTTTGCC	40	20	
F3	ACCAGCTACGCAGCACA	17	20	
B3	CACTTGATTCGTTACCAGTGAATAGG	26	20	
LF	GCAACGGTTGCTTTCGG	17	20	
LB	GTTTGATCATGAAGTCTGTGG	21	20	
Extra primer				This study
FIP2 (F1c-2 + F2)	TGGTGTTAGACGGAATTCTTTTCGATCAATGAGTACGGCTACGA	44	20	
BIP2 (B1c-2 + B2)	CACCTAGTGAACGAACTTCTTTTCGATTTCTTC**CC**CATTTTGCC	44	20	
F1c-2	TGGTGTTAGACGGAATTC	18	20	
B1c-2	CACCTAGTGAACGAACTTC	19	20	

The underlines indicate the T-T-T-T linkers. The bold letters indicate the modified bases to reduce hair-pin structures.

### MHP-LAMP assay

LAMP assay was adopted from the standard LAMP protocol suggested by New England Company Ltd. (https://international.neb.com/protocols/2014/06/17/loop-mediated-isothermal-amplification-lamp). Its reaction was carried out in a final volume of 25 µL containing 1× ThermoPol-supplied reaction buffer (20 mM Tris-HCl, 10 mM (NH_4_)_2_SO_4_, 10 mM KCl, 2 mM MgSO_4_, 0.1% Triton X-100, pH 8.8), 0.8 M betaine (Sigma-Aldrich, MO, USA), 6 mM MgSO_4_ (New England Biolabs, MA, USA), 1.6 mM dNTP mix (New England Biolabs, MA, USA), and 8 U *Bst* 2.0 WarmStart DNA polymerase (New England Biolabs, Ipswich, MA), each *rpoD* primer set (Table 2), and 1 µL of gDNA template*.* The final volume was adjusted to 25 µL using UltraPure^TM^ DW (Invitrogen, Grand Island, Germany). The negative control containing no gDNA was included in each run. LAMP reaction was done in Loopamp Realtime Turbidimeter LA-320C (Eiken Chemical Co Ltd, Tokyo, Japan) at 65 °C, 75 min followed by DNA polymerase inactivation at 80 °C for 5 min. The results were reported in a real-time amplification plot format and were inspected using 3% agarose gel electrophoresis (AGE).

**Table 2:** Primer combinations and their efficiency in boosting LAMP detection sensitivity by means of detection limit (DL) and speed determination.

Primer	*rpoD* ref. primer set concentration (pmol/µL)	Combination I concentration (pmol/µL)	Combination II concentration (pmol/µL)	Combination III concentration (pmol/µL)
FIP1	40	20	40	20
FIP2	–	20	–	20
BIP1	40	20	40	20
BIP2	–	20	–	20
LF	40	40	20	20
LB	40	40	20	20
F1c-2	–	–	20	20
B1c-2	–	–	20	20
F3	10	10	10	10
B3	10	10	10	10
Detection sensitivity in the unit of gDNA copies/reaction extracted from Fig. 2	10^3^	10^3^	10^3^	10^2^
Detection speed (min) of 10^3^ copies/reaction	47	40	39	33 (∼30% faster than the reference primer set)

### Evaluation of the sensitivity and the specificity of MHP-LAMP assay

The newly designed extra MHPs were combined with the original *rpoD* core primers [14] in various combinations (Set I, II, and III) (Table 2) prior to being tested using 10^4^ to 10 copies/reaction of reference strain gDNA as the templates. Each combination was performed in triplicate. The best-fit primer combination was further tested for its specificity using a number of pathogens as templates. Of 17 samples tested, 3 were *V. parahaemolyticus* reference strains, 11 were other pathogens, of which 3 were RNA viruses (Table 3).

**Table 3:** Specificity of the MHP-LAMP assay using the primer combination Set III targeting *rpoD* gene. All samples tested here were the left overs from our previous study [19].

Organism		Number of	Result by turbidity
		isolates	measurement and AGE
Target	Gram negative		
Bacteria	*V. parahaemolyticus* reference strains	3	+
Non-target	Gram negative		
Bacteria	*Aeromonas veronii*	1	-
	*Aeromonas jandaei*	1	-
	*Aeromonas caviae*	1	-
	*Aeromonas hydrophila*	1	-
	*Edwardsiella tarda*	1	-
	*Flavobacterium columnare*	1	-
	*Francisella orientalis*	1	-
	*Hahella chejuensis*	1	-
	*Pseudomonas aeruginosa*	1	-
	*Vibrio cholerae*	1	-
	*Vogesella sp.*	1	-
	Gram positive		
	*Staphylococcus aureus*	1	-
	*Streptococcus agalactiae*	1	-
	*Steptococcus iniae*	1	-
Non-target	Virus		
Virus	Scale Drop Disease Virus (SDDV)	1	-
	Tilapia Lake Virus (TiLV)	1	-
	Total	17	

## Method validation

### Principle of conventional LAMP and MHP-LAMP

**Fig. 1:**
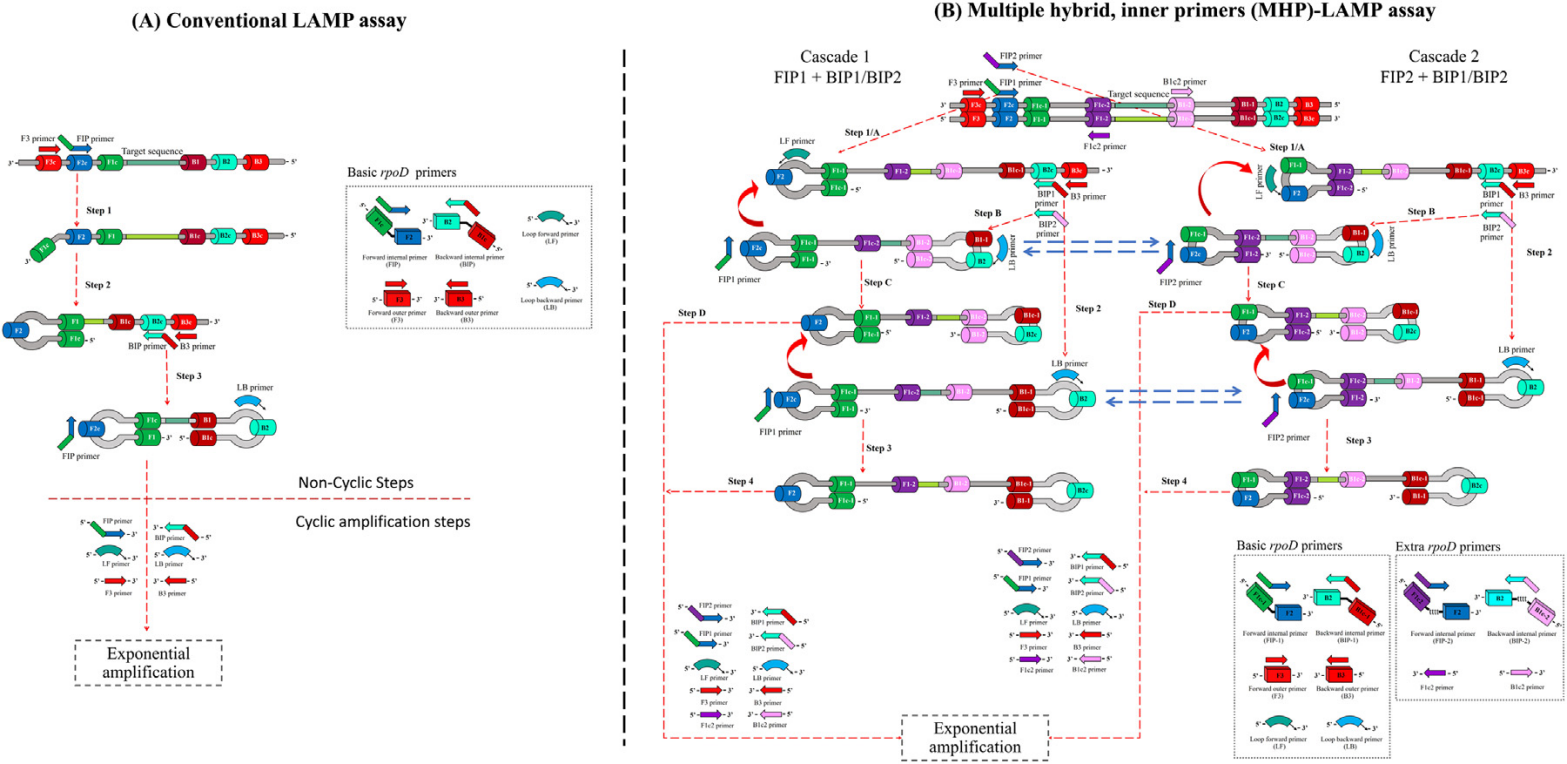
Schematic diagram of 2 different LAMP stratagies demonstrated in this study. (A) Conventional LAMP assay with *rpoD* basic primers. (B) Multiple hybrid, inner primer (MHP)-LAMP assay with *rpoD* basic primers *and* extra inner primers.

In conventional LAMP, two inner primers (FIP and BIP), and two outer primers (F3 and B3) are required for the detection of *V. parahaemolyticus rpoD* gene (Fig. 1A). Its mechanism consists of 2 major steps: (1) non-cyclic step that generates stem loop DNA with a dumbbell-shaped structure which serves as a template for the later step, and (2) cyclic amplification step that synthesizes LAMP amplicons exponentially (Fig. 1A). To begin with, the FIP hybridizes to F2c region of *rpoD* gene and initiates DNA synthesis via the strand displacement activity of *Bst* DNA polymerase. Subsequently, the F3 hybridizes to F3c region and initiates strand displacement DNA synthesis to release a single strand of FIP-linking complementary strand which forms a stem-loop structure at 5′ end. The BIP hybridizes to B2c region of the FIP-linking complementary strand and initiates DNA synthesis. Later, the B3 hybridizes to B3c region and initiates strand displacement DNA synthesis to release a single strand of the BIP-linking complementary strand which forms a structure with stem-loop at each end, which resembles to a dumbbell structure. This structure is the template for LAMP auto-cycling amplification steps. To accelerate LAMP reaction, loop primers forward (LF) and backward (LB) binding to the single-stranded loop region (between F1 and F2 and between B1 and B2) are used. These loop primers help to increase the numbers of starting points for DNA synthesis.

In MHP-LAMP, four newly designed extra primers, which targeted 4 more distinct regions (F1c-1, F1c-2, B1c-1, B1c-2), were utilized. These included two hybrid inner primers (FIP2 and BIP2) and two loop-like primers (F1c-2 and B1c-2). The FIP2 consists of sequences of F1c-2, a T-T-T-T linker, and F2, while the BIP2 consists of sequences of B1c-2, a T-T-T-T linker, and B2 (Table 1). Note that the FIP2 and BIP2 share the upstream stretch with the FIP1 (F2) and BIP1 (B2), respectively. This design was intended due to the limited availability of appropriate, unoccupied sequence in between F2 and B2 regions (Fig. S1). The FIP1, FIP2 and F3 hybridize the DNA target and prime the DNA synthesis displacement, producing FIP1-linking complementary strand DNA and FIP2-linking complementary strand DNA, each containing a stem-loop structure at the 5′-end (Fig. 1B). Both DNA strands serve as the templates for strand displacement DNA synthesis primed by BIP1, BIP2 and B3 resulting in four dumbbell-shaped structures with different lengths which quickly convert into stem-loop DNA structures, the starting structure for LAMP auto-cycling strand displacement DNA synthesis. In LAMP auto-cycling steps, FIP1 and FIP2 hybridize with the complementary loops and prime the strand displacement DNA synthesis. The recently synthesized DNA eventually conducts self-primed DNA synthesis. The BIP1 and BIP2 subsequently hybridize to the corresponding loops and generate more double strand DNAs with loops. More double strand DNAs are produced as the reaction continues. In summary, from non-cyclic amplification, the MHP-LAMP produces four different dumbbell-shaped DNA templates to serve cyclic amplification, while the conventional LAMP does only one. This significant difference led to the hypothesis that the MHP-LAMP would have higher efficiency than the conventional LAMP in terms of sensitivity and speed. Tests to prove this hypothesis were carried out in the next section.

### Design and validation of primer combinations in enhancing MHP-LAMP assay's sensitivity and speed

**Fig. 2:**
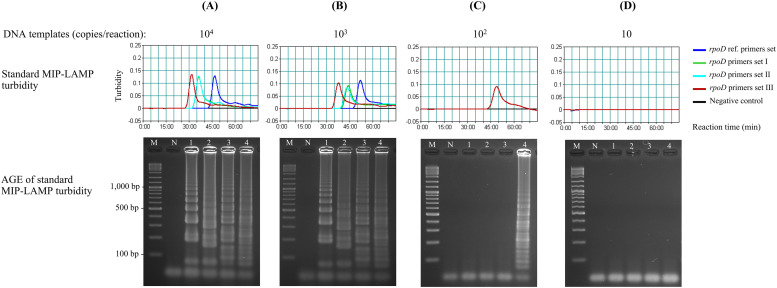
Comparative detection sensitivity and speed of MHP-LAMP assay run on various concentrations of gDNA templates (10^4^ – 10 copies/reaction), and various primer combinations (Set I, II, III) against the reference core *rpoD* primer set. The test results were analyzed by real-time turbidity measurement of magnesium pyrophosphate (Top) and agarose gel electrophoresis (Bottom). M: 1 kb DNA ladder; N: Negative control, Lanes 1: core *rpoD* reference primers. Lanes 2-4: *rpoD* primer combination Set I, II, III, respectively. Clearly, the combined primer Set III has the highest detection sensitivity as demonstrated by the lowest limit of detection at 100 copies.

To determine whether our extra primers was able to boost up conventional LAMP amplification sensitivity and speed, separated groups of LAMP reactions were performed with various primer combinations (I, II, III) against the reference core *rpoD* primer set by using various concentrations of gDNA as templates: 10^4^ – 10 copies/reaction, (Table 2). The reactions were incubated at 65 °C for 75 min and LAMP product signals were detected by Loopamp Realtime Turbidimeter at 650 nm wavelength. The data was exhibited as the signal depicting the amplified product of each time point at real-time which the turbidity peaked and then dropped with time when the LAMP substrate was used up. No amplification signal was observed as expected for a negative control up to 75 min (Fig. 2, black line). All 4 primer combinations showed positive amplifications at 10^4^ and 10^3^ copies/µL and showed a dose response effect in which the higher concentration of DNA showed a faster appearance of peak signals, corresponding to the accumulation of amplified products (Fig. 2). Set III showed the fastest amplification signal (red line), followed by Set II (light blue line) and I (green line), and conventional core primers (deep-blue line), respectively. To illustrate, at 10^3^ copies/µL (Fig. 2B), Set III started showing the positive signal at 33 min or approximately 30 % earlier than the conventional set that began to show at approximately 47 min. Most importantly, Set III was the only combination that detected the target DNA down to 10^2^ copies (Fig. 2C), highlighting that it was not only enhance the speed and also the detection sensitivity as hypothesized. We believe that this phenomenon was due to the synergistic effect of core primers and extra primers added into the system (see Fig. S2 for further discussion).

### Validation of MHP-LAMP specificity

In MHP-LAMP assay with the primer combination Set III, positive amplification was only observed in *V. parahaemolyticus* gDNA samples indicating that this method was highly specific for *V. parahaemolyticus* detection (Table 3).

## Discussion and conclusions

In this study, a novel molecular method named “MHP-LAMP” has been developed to identify *V. parahaemolyticus*, one of major food-borne pathogens [15]. The method was built on the conventional LAMP assay [14] by the addition of two newly designed extra primers (FIP2 and BIP2) and two extra loop-like primers (F1c-2 and B1c-2) to enhance the assay efficiency. Each type of primers functions differently. The former hybridizes to the target directly to produce more starting materials for the cyclic amplification procedure, thus increasing the reaction rate and improving the detection sensitivity [16,17]. The latter binds the unoccupied loop-adjacent regions to facilitate multiple DNA displacement, that aids in boosting up the reaction speed [18]. The MHP-LAMP detected *rpoD* gene as low as 100 copies/reaction in 44 min, namely 10 times more sensitive than the conventional LAMP whose detection limit (DL) was 1,000 copies/reaction at 47 min incubation onwards. Further incubation up to 75 min did not enhance the DL of both assays. It is worth noting that at 1,000 copies DNA, the MHP-LAMP took only 33 min to produce positive signal which was faster than the conventional LAMP by 30% (Table 2). Although this finding is not beneficial to the end users since incubating at least 44 min is still recommended to maximize the detection sensitivity (100 copies/reaction), it serves as evidence to prove that our concept of using extra primers to enhance LAMP diagnostic performance is correct and valid. This concept can be applied to boost up sensitivity and rapidity of other existing LAMP-based assays. It helps to reduce the cost and time to redesign whole new sets of primers from scratch. In food sample analysis, the MHP-LAMP technique has the potential to be fast, sensitive, and specific for *V. parahaemolyticus* detection.

## Declaration of Competing Interest

The authors declare that they have no known competing financial interests or personal relationships that could have appeared to influence the work reported in this paper.

## Data Availability

Data will be made available on request.

## Appendix Supplementary materials

Image, application 1
