# Supplementary material for: Enhancement of loop mediated isothermal amplification's sensitivity and speed by multiple inner primers for more efficient identification of Vibrio parahaemolyticus
Source: MethodsX. 2023 Aug 21;11:102328. doi: 10.1016/j.mex.2023.102328 (PMC10491634; doi:10.1016/j.mex.2023.102328)
Supplement: Supplementary file 1 [file mmc1.docx]

**Supplementary information**

**Article title**

Enhancement of loop-mediated isothermal amplification’s sensitivity and speed by multiple inner primers for more efficient identification of *Vibrio parahaemolyticus*

**Authors**

Aekarin Lamalee^1^*^a^*, Chartchai Changsen^1^*^a^*, Wansadaj Jaroenram^2^, Sureemas Buates^1*^

***^a^*These authors contributed equally to this work.**

**Affiliations**

^1^Department of Microbiology, Faculty of Science, Mahidol University, Bangkok 10400, Thailand.

^2^Bioengineering and Sensing Technology Research Team, National Center for Genetic Engineering and Biotechnology (BIOTEC), National Science and Technology Development Agency (NSTDA), Pathum Thani 12120, Thailand


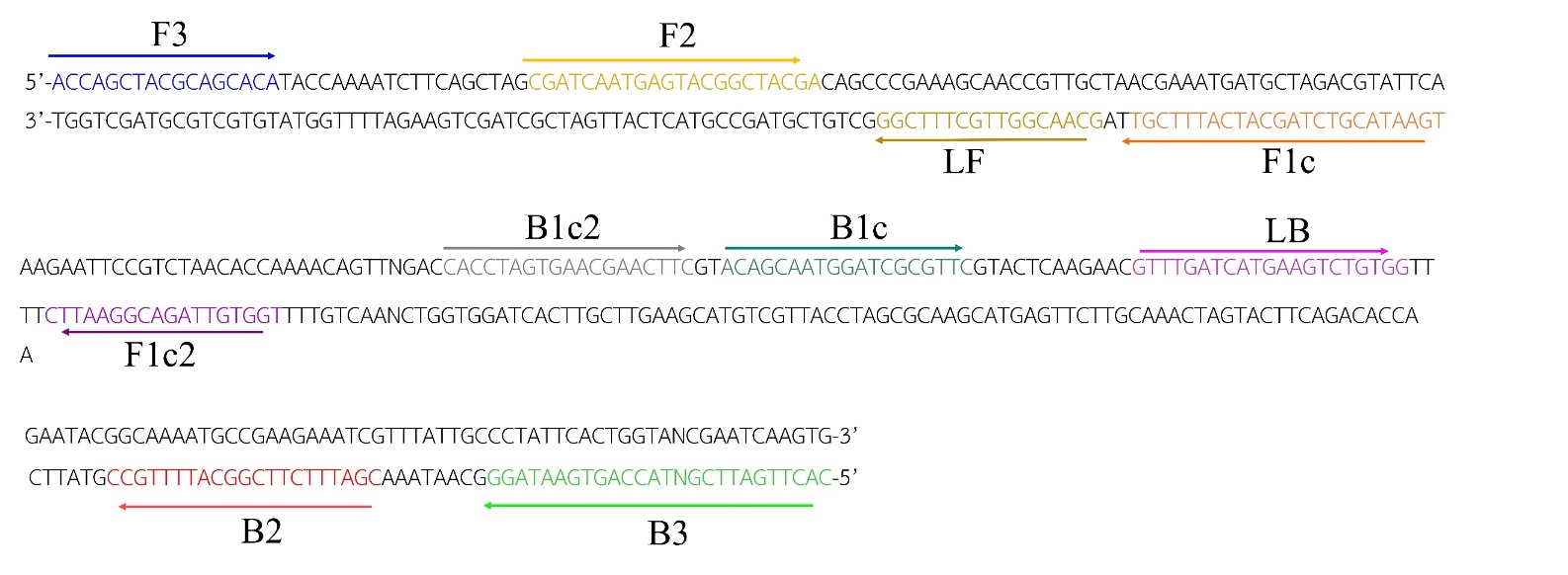


**Fig. S1.** Location of *V. parahaemolyticus* specific primers used in this study with their nucleotide sequences. The conventional LAMP consists of 6 cores primers designated as F3, B3, FIP1 (F1c/TTTT/F2), BIP1 (B1c/TTTT/B2), LF and LB. For the MHP-LAMP assay (this study), four additional primers [FIP2 (F1c-2/TTTT/F2), BIP2 (B1c-2/TTTT/B2), F1c and F1c-2] were incorporated between the typical FIP and BIP regions to maximize the LAMP kinetics and to sensitively detect *V. parahaemolyticus*. Arrows indicate the direction from 5′ to 3′ ends. Primer sequences are listed in [Table 1](#Table1).


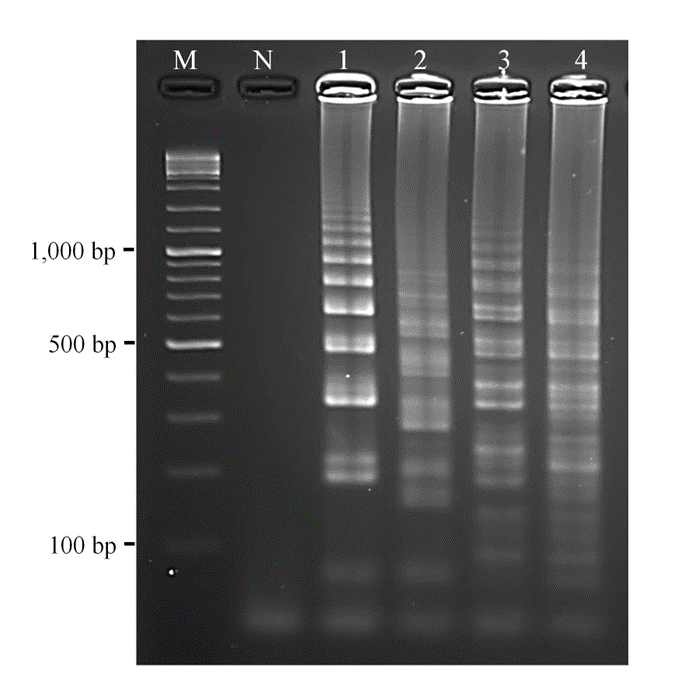


**Fig. S2.** Patterns of LAMP amplicons obtained from MHP-LAMP amplification using different combinations of primers listed in Table 2. Lane M: 1kb DNA ladder; lane N: negative control. Lane 1: *rpoD* reference primer (no combination with other extra primers). Lanes 2-4: primer combinations Set I-III, respectively. Obviously, the combination Set III resulted in the highest variations in sizes of LAMP amplicons. This is highly likely due to the highest numbers of primers added, and the synergy of them. This finding was in accordance with the highest degree of detection sensitivity the primer Set III revealed, highlighting that our hypothesis that “the addition of extra inner primers (FIP2, BIP2, F1c-2, B1c-2) can enhance sensitivity and speed of LAMP assay” was correct.

**Reference**

[1] J. Nemoto, C. Sugawara, K. Akahane, K. Hashimoto, T. Kojima, M. Ikedo, H. Konuma, Y. Hara-Kudo, Rapid and specific detection of the thermostable direct hemolysin gene in *Vibrio parahaemolyticus* by loop-mediated isothermal amplification, J. Food Prot. 72 (2009) 748-754, doi:[10.4315/0362-028x-72.4.748.](https://doi.org/10.4315/0362-028x-72.4.748)
